# Postpartum Septic Pelvic Thrombophlebitis in a Tertiary Maternity Hospital in Dubai, UAE

**DOI:** 10.7759/cureus.36452

**Published:** 2023-03-21

**Authors:** Elham A Akbari, Rawan Majdalawi, Deemah K Harb, Komal Hazari, Widad Abdelkareem, Abeir Ammar

**Affiliations:** 1 Obstetrics and Gynecology, Latifa Hospital - Dubai Academic Health Corporation, Dubai, ARE; 2 Internal Medicine, Latifa Hospital - Dubai Academic Health Corporation, Dubai, ARE; 3 Obstetrics and Gynaecology, Latifa Hospital - Dubai Academic Health Corporation, Dubai, ARE

**Keywords:** venous thromboembolism (vte), postpartum abdominal pain, ovarian vein thrombosis, septic pelvic thrombophlebitis, postpartum fever

## Abstract

Septic pelvic thrombophlebitis (SPT) is a well-known condition, yet it remains a rare postpartum complication. It can be divided into two types: deep septic pelvic thrombophlebitis (DSPT) and ovarian vein thrombosis (OVT).

In this case series, we present three cases diagnosed with ovarian vein thrombosis that were managed in our tertiary care hospital, Latifa Women and Children Hospital (LWCH), in Dubai, UAE. It is a 440-bed public tertiary care center that specializes in maternal and neonatal services, with a range of 3500 to 4000 deliveries per year. The three cases represent the total number diagnosed with this condition in the period between 2018 and 2022 among the total obstetric population during this period.

The three cases developed a fever in the postpartum period, which for several days did not respond to the standard antibiotics used for endometritis. Two cases were following a cesarean section, and the third case was following vaginal delivery complicated with severe postpartum hemorrhage and hysterectomy. The clinical suspicion and awareness of the condition paved the way to reach the proper diagnosis and initiate the therapeutic dose of anticoagulants, along with broad-spectrum antibiotics, in a timely manner.

﻿The prompt diagnosis with early intervention led to optimal patient outcomes and prevented the morbidity and mortality associated with this condition.

## Introduction

Postpartum ovarian vein thrombosis (POVT) is a rare pregnancy complication that can result in life-threatening consequences such as pulmonary embolism and sepsis [1,2]. It usually occurs between day 2 and day 14 post-delivery and can lead to significant morbidity and mortality [1].

The incidence of symptomatic ovarian vein thrombosis (OVT) in the postpartum period ranges between 0.01% and 0.05% of all pregnancies (1 in 3000 deliveries). Literature has shown that OVT occurs in one in 9000 vaginal deliveries and one in 800 cesarean sections. These percentages reflect that cesarean sections and gynecological surgeries have a higher risk as compared to vaginal deliveries [3-5]. In our case series, it had occurred after a cesarean section and a vaginal delivery that was complicated by a hysterectomy.

Our case series describe the clinical presentation, laboratory findings, imaging findings, management, course of the illness, and the follow-up of three cases diagnosed with ovarian vein thrombosis in the postpartum period.

## Case presentation

Case 1

A 40-year-old, multi gravida, para 6, presented on day 10 post-emergency cesarean section with right lower abdominal pain associated with fever and vomiting for one week. She sought consultation in another facility and was treated for three days with intravenous antibiotics (IV), including ceftriaxone 1000 mg every 12 hours and metronidazole 500 mg every eight hours, but as she remained febrile and symptomatic, she presented to our emergency department for further management.

Upon arrival at the emergency department, the patient was vitally stable. On examination of the abdomen, a tender vague mass was palpable in the right lumbar and iliac regions associated with mild suprapubic tenderness. Per speculum examination revealed cervical motion tenderness with adnexal fullness associated with tenderness on the right side. The left adnexa was free. Lab investigations were ordered, which showed a mild rise in the C-reactive protein (CRP) (Table 1).

**Table 1:** Investigations - Case 1

	Admission (day 17 post-delivery)	Day 4 of admission
White Blood Cell count (10^3/uL)	6.2	5.6
C-Reactive Protein (mg/L)	69.8	19.2
Procalcitonin (ng/mL)	0.04	0.03
Hemoglobin (g/dL)	9.7	11.3
D-Dimer (ug/mL FEU)	Not done	Not done
Cultures (blood + urine + high vaginal swab (HVS))	Negative	
Thrombophilia Screening (acquired and heritable) after stopping anticoagulants	Negative	

Upon admission, she started spiking fever and broad-spectrum IV antibiotics were initiated along with a prophylactic dose of enoxaparin. Pelvic ultrasound showed evidence of a heterogeneous soft tissue mass lesion approximately measuring 5.6 x 3 cm raising the possibility of an oedematous right fallopian tube suggestive of right adnexal mass with a probability of salpingitis.

**Figure 1:**
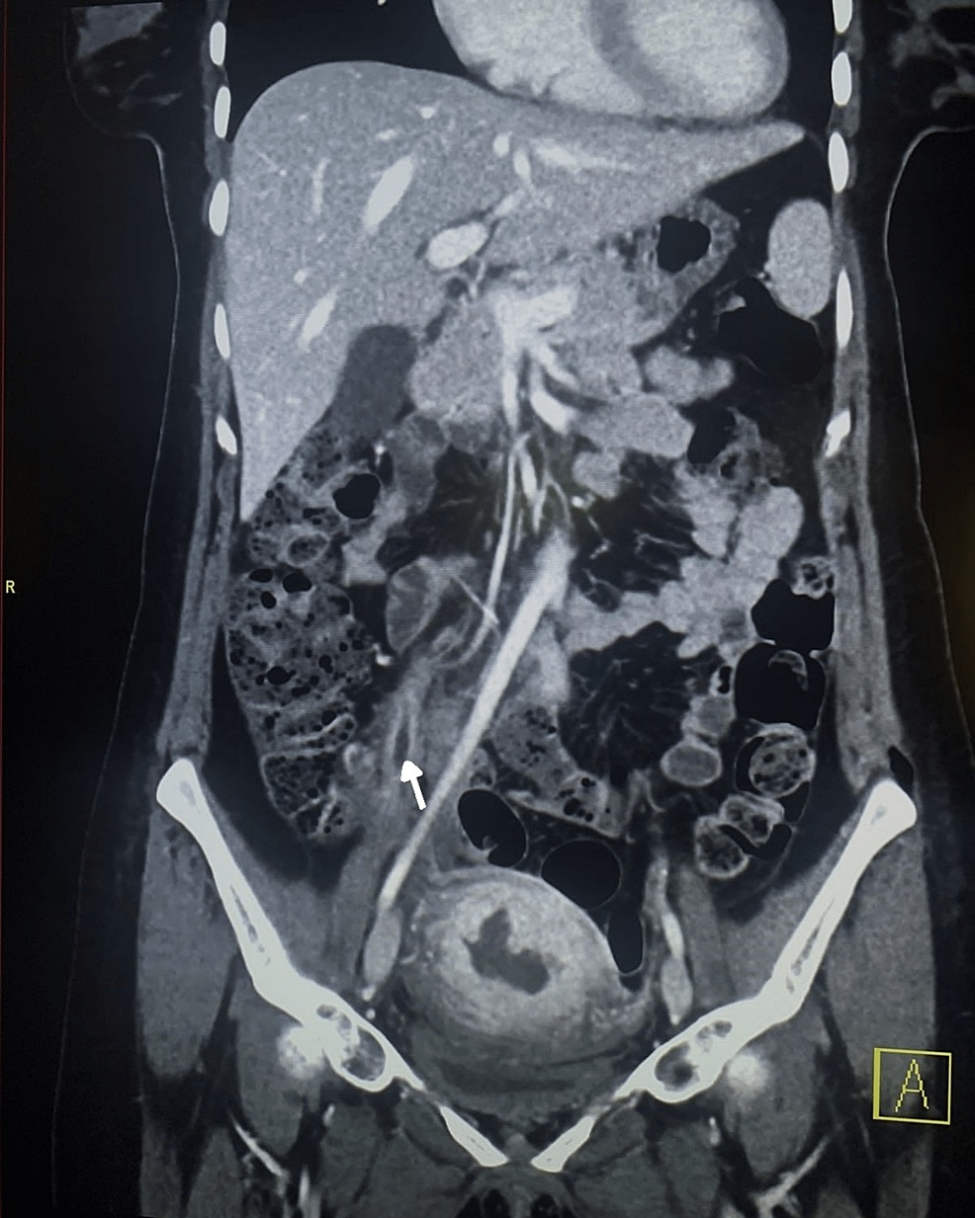
CT scan of the pelvis in Case 1 showing right ovarian vein thrombosis (arrow)

We proceeded with diagnostic laparoscopy on day 1 of admission, which revealed right ovarian vein thrombosis (OVT) with no evidence of intraperitoneal infection. Enoxaparin was increased to a therapeutic dose (1 mg/kg every 12 hours ). A computed tomography (CT) scan of the abdomen and pelvis with contrast was done on day 3 of admission confirming the diagnosis of right ovarian vein thrombosis along with features of endometritis (Figure 1).

The patient received IV antibiotics for a total of seven days. Her clinical condition showed dramatic improvement after commencing the anticoagulants and was discharged home on day 8 in stable condition on a therapeutic dose of enoxaparin.

A follow-up CT scan after two months was suggestive of chronic thrombophlebitis of the right ovarian vein. She received anticoagulants for a total of six weeks.

Case 2

A 38-year-old, multi-gravida para 8, presented to our emergency department on day 17 post-vaginal delivery with complaints of fever for one week. Her delivery was complicated by a severe postpartum hemorrhage and needed a hysterectomy. One week later, the patient developed a fever and was admitted to another facility with the diagnosis of infected pelvic hematoma and was started on broad-spectrum antibiotics for three days, but as she was still spiking fever, she decided to present to our facility for further advice.

Upon arrival, she was vitally stable and her abdomen was soft on palpation with mild tenderness over the hysterectomy incision scar. Lab investigations were ordered, which showed mild leucocytosis, raised procalcitonin level, raised C-reactive protein level, and raised D-dimer (Table 2).

**Table 2:** Investigations - Case 2

	Admission (day 17 post-delivery)	Day 2	Day 4
White Blood Cell count (10^3/uL)	12.3	10.8	11.8
C-Reactive Protein (mg/L)	153	123	55.7
Procalcitonin (ng/mL)	2.5	1.02	0.43
Hemoglobin (g/dL)	9.7	9.6	9.9
D-Dimer (ug/mL FEU)	3.28	Not done	Not done
Cultures (blood + urine + HVS)	Negative		
Thrombophilia screening (acquired and heritable) after stopping anticoagulants	Negative		

**Figure 2:**
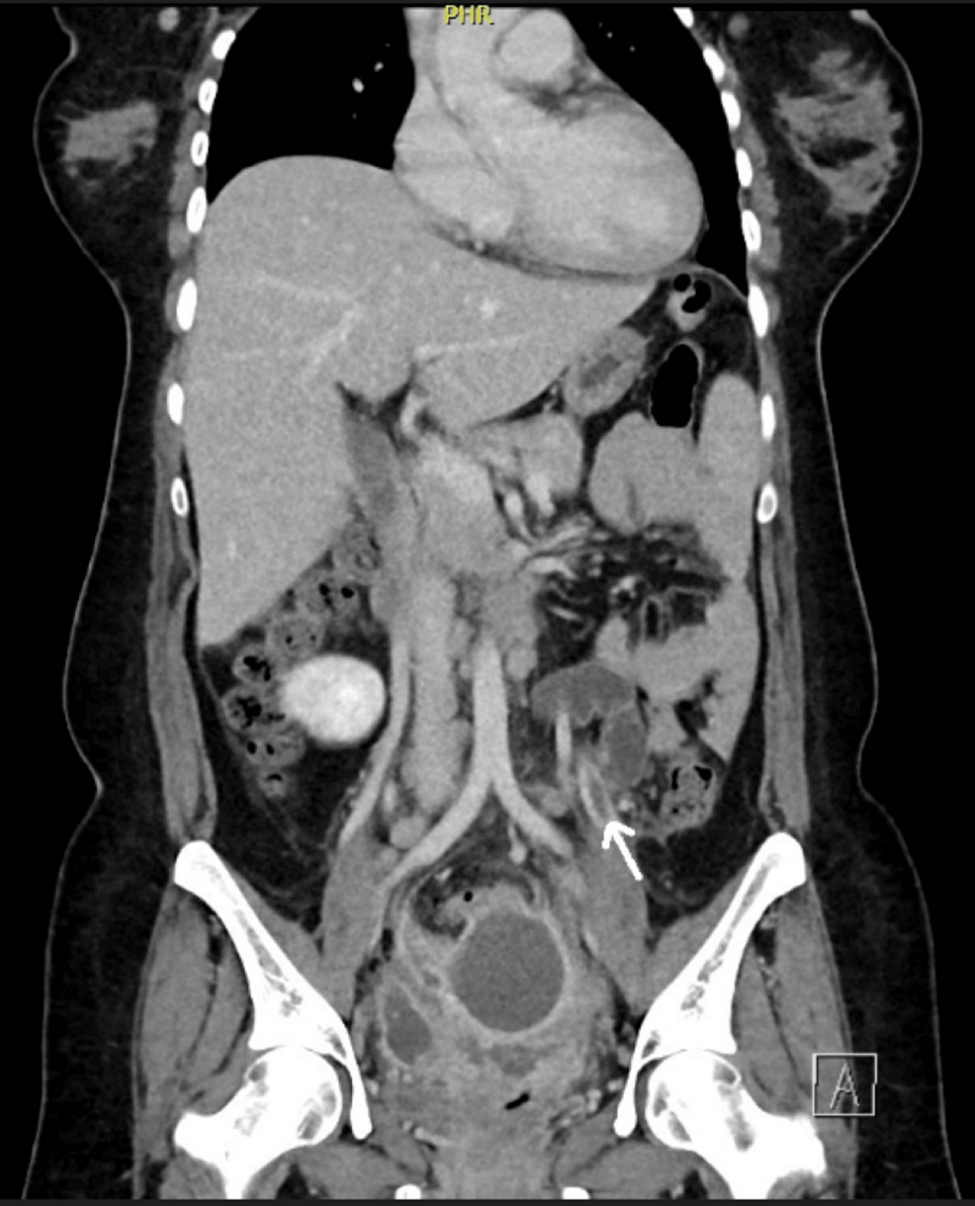
CT scan of the pelvis in Case 2, showing left ovarian vein thrombosis (arrow)

After admission, she was spiking a fever of 38 to 38.5 degree Celsius. Broad-spectrum IV antibiotics were commenced along with a prophylactic dose of enoxaparin. With suspicion of septic pelvic thrombophlebitis (SPT), she was scheduled for a CT scan of the abdomen and pelvis on the same day of admission, which confirmed the diagnosis of left ovarian vein thrombosis with partial thrombosis of the right ovarian vein at its proximal end (Figure 2).

The enoxaparin dose was increased to a therapeutic dose. The patient remained stable and showed a good response. Within 48 hours, she became afebrile with resolving abdominal pain, and gradually her symptoms subsided. Hence, she was discharged home on day 5 in stable condition on a therapeutic dose of enoxaparin.

She received therapeutic enoxaparin for eight weeks. The follow-up CT scan after two months was normal, and anticoagulants were stopped.

Case 3

A 19-year-old, primigravida, was admitted to the delivery suite at 37 weeks in early labor. During the intrapartum period, she spiked a fever and was started on IV antibiotics for a suspected intra-amniotic infection. The patient underwent an emergency cesarean section at full dilatation due to failure to progress. Postoperatively, she was on a prophylactic dose of enoxaparin and continued on IV antibiotics. She was still running a fever, hence IV antibiotics were escalated. Despite that, she continued to be febrile, ranging between 37.8 and 38.6-degree C, along with rising inflammatory markers (Table 3).

**Table 3:** Investigations - Case 3

	Intrapartum	Day 1	Day 3	Day 5
C-Reactive Protein (mg/L)	44.5	182	121	89.7
Procalcitonin (ng/mL)	0.17	7.62	2.96	1.46
White Blood Cell count (10^3/uL)	13.2	14.0	9.8	8.9
D-Dimer (ug/mL FEU)	-	8.7		2.66
Cultures (blood + urine + HVS)	Negative			

**Figure 3:**
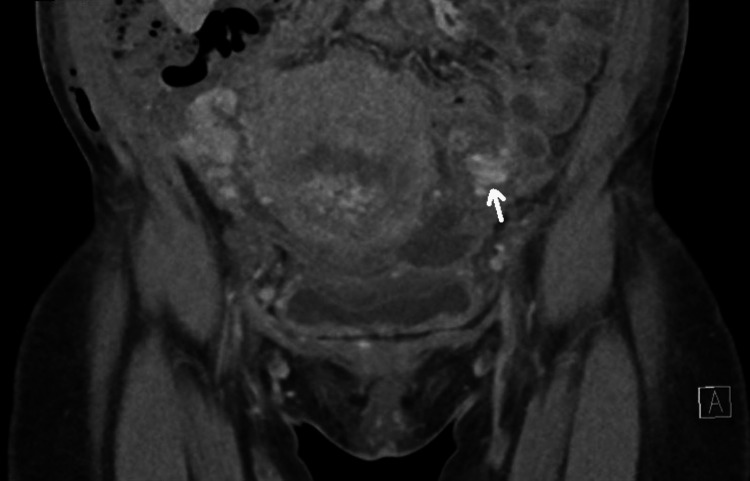
CT scan of the pelvis in Case 3 showing left ovarian vein thrombosis (arrow)

On day 5 postoperatively, the patient started complaining of left lower abdominal pain associated with chills and rigors. On examination, the patient was vitally stable, and the rest of the systemic examination was not significant. With the suspicion of SPT, therapeutic enoxaparin was started and a CT scan of the abdomen and pelvis was scheduled on the next day, which confirmed the diagnosis of left ovarian vein thrombosis (Figure 3).

The patient's clinical condition improved dramatically after starting a therapeutic dose of enoxaparin. She remained afebrile and symptom-free and was discharged two days later in stable condition on a therapeutic dose of enoxaparin. Unfortunately, she defaulted, and we lost follow-up.

Table 4 summarizes the above three cases.

**Table 4:** Summary of all cases GA: gestational age; VTE: venous thromboembolic event

CASE	1	2	3
Age (years)	40	38	19
BMI	24.4	28.4	22.6
Parity	6+3	8+1	0+0
GA at delivery (weeks)	40	38	37
Mode of delivery	Emergency cesarean section	Vaginal delivery complicated by hysterectomy	Emergency cesarean section
Symptoms and vitals	Fever, right lower abdominal pain, vomiting	Fever	Fever, chills, rigors, left lower abdominal pain
Onset of symptoms from the day of delivery	10	10	Intrapartum
Personal history of VTE	nil	nil	nil
Family history of VTE	nil	nil	nil

## Discussion

The pathophysiology of venous thromboembolic events (VTEs) in general, including ovarian vein thrombosis in pregnancy and the postpartum period, can be explained by Virchow’s triad, due to the hypercoagulable state in pregnancy, compression of the inferior vena cava by the gravid uterus, which causes venous stasis, and the inflammation or injury to the endothelium during delivery [3].

Studies have shown that the right ovarian vein is involved more compared to the left and that occurs mainly due to the compression of the right ovarian vein and inferior vena cava due to dextrorotation of the gravid uterus, and in addition to it having a longer course, narrower lumen with incompetent valves [6]. However, in our case series, two out of the three cases were diagnosed with left ovarian vein thrombosis.

Commonly, the patient present within the first two weeks of delivery with lower abdominal or flank pain. It can also be associated with fever and leucocytosis [3]. The fever could have a spiking pattern that fails to respond to the standard broad-spectrum antibiotic therapy. Furthermore, the patient usually does not appear toxic. The finding of a tender abdominal mass described as rope or sausage-shape is considered a diagnostic yet rare clinical finding [7]. Our patients shared some of the signs and symptoms of fever, leucocytosis, and vague abdominal pain but none had elicited the specific rope-like mass.

The diagnosis is challenging since the clinical findings are non-specific [8]. Ultrasound imaging is not reliable; however, it can aid in excluding other differential diagnoses such as deep/space surgical site infections. With the help of advanced imaging modalities, such as CT or magnetic resonance imaging (MRI), diagnosis has become more achievable [7,8].

In our cases, ultrasound imaging failed in reaching the diagnosis. However, a pelvic CT scan aided in the proper diagnosis of OVT. The preferred imaging technique is MRI, as it offers better visualization of soft tissue changes and is able to evaluate the resolution of edema and inflammatory signs [7].

The treatment consists mainly of anticoagulation with or without broad-spectrum antibiotics depending on the features of a coexisting infection [7]. In our cases, all had received antibiotics prior to presenting to us with an average duration of three to five days. In view of the raised inflammatory markers, we did combine anticoagulants with continuing the broad-spectrum antibiotics regimen used for endometritis although all the obtained cultures were negative. Antibiotics were stopped after 48 hours from being afebrile and showing clinical improvement while anticoagulants continued for eight weeks in two cases while the third one was lost to follow-up.

Furthermore, in reviewing our patients' course of management, it can be noticed that they didn’t show improvement with antibiotics prior to starting anticoagulants. In comparison, when observing their clinical condition after commencing the therapeutic dose of enoxaparin, dramatic improvement was observed within 24 to 48 hours.

Anticoagulation's role is to prevent the progression of thrombosis, prevent embolization, and reduce the extension of the septic emboli. The duration is still debated in the literature with no studies confirming optimal duration. The resolution of postpartum ovarian vein thrombosis (POVT) has been documented after only seven to 14 days of treatment; however, others consider giving the treatment for three to six months. As there is an acute cause for POVT, long-term anticoagulation is not recommended unless the patient is having the following risk factors: embolic disease outside the pelvis, chronic pro-thrombotic risk factors (thrombophilia disorders), or hypercoagulability (e.g., related to malignancy) [3,6,8-12].

Morbidity in POVT is due to two of the most serious maternal-related complications including a systemic extension of the clot and sepsis. Pulmonary embolism estimated risk is 25% of the cases, with 4-5% overall estimated mortality, accounting for much of the 4-5% overall mortality [8,10,13,14]. Other complications of POVT include ovarian abscess, ovarian infarction, uterine necrosis, and sepsis. However, most patients recover without significant morbidity.

The recurrence rate of POVT is unknown, but ovarian vein thrombosis from all causes may recur at rates similar to deep vein thrombosis [14-16].

Moreover, considering individual risk factors by strictly implementing the venous thromboembolism (VTE) risk assessment score in our obstetric population, it is advisable to consider septic pelvic thrombophlebitis (SPT) as an adding VTE risk factor in those populations where more studies are needed to establish a clear recommendation [3,10].

## Conclusions

Although SPT remains a rare condition, it may be on the rise since we have an increasing number of cesarean sections performed every day. Postpartum septic pelvic thrombophlebitis is an important and challenging differential diagnosis that physicians should be aware of, especially when managing patients presenting with postpartum fever and abdominal pain not responding to the standard broad-spectrum antibiotics recommended for the coverage of endometritis in the absence of an alternative diagnosis. The treatment consists mainly of therapeutic dose anticoagulation with or without broad-spectrum antibiotics depending on the features of a coexisting infection. The optimal duration of anticoagulation therapy in SPT remains uncertain; in OVT, the recommended anticoagulation duration is up to six weeks and long-term anticoagulation is recommended in patients with co-existing prothrombotic risk factors. It is advisable to consider septic pelvic thrombophlebitis (SPT) as an adding VTE risk factor in those populations where more studies are needed to establish a clear recommendation. The need for prophylactic anticoagulation in the future pregnancies of these patients is still lacking clinical evidence but considering the rarity of the condition and the related complications, we believe, as clinicians, that it may be justified to consider initiating the anticoagulation prophylaxis at an advanced stage of gestation (from 28 weeks).

Our aim is that this case series will aid physicians who are involved in the management of persistent post-partum fever to become more aware of the condition's presentation, complications, and course of management. Prompt diagnosis and treatment can not only prevent potentially life-threatening complications but also reduce the prolonged hospital stay days and the extended course of the illness.
